# Who is at risk of lung nodules on low-dose CT in a Western country? A population-based approach

**DOI:** 10.1183/13993003.01736-2023

**Published:** 2024-06-06

**Authors:** Jiali Cai, Marleen Vonder, Yihui Du, Gert Jan Pelgrim, Mieneke Rook, Gerdien Kramer, Harry J.M. Groen, Rozemarijn Vliegenthart, Geertruida H. de Bock

**Affiliations:** 1 Department of Epidemiology, University of Groningen, University Medical Center Groningen, Groningen, The Netherlands; 2 Department of Epidemiology and Health Statistics, School of Public Health, Hangzhou Normal University, Hangzhou, China; 3 Department of Radiology, University of Groningen, University Medical Center Groningen, Groningen, The Netherlands; 4 Department of Radiology, Medisch Spectrum Twente, University of Twente, Enschede, The Netherlands; 5 Department of Radiology, Martini Hospital Groningen, Groningen, The Netherlands; 6 Department of Pulmonology, University of Groningen, University Medical Center Groningen, Groningen, The Netherlands

## Abstract

**Background:**

This population-based study aimed to identify the risk factors for lung nodules in a Western European general population.

**Methods:**

We quantified the presence or absence of lung nodules among 12 055 participants of the Dutch population-based ImaLife (Imaging in Lifelines) study (age ≥45 years) who underwent low-dose chest computed tomography. Outcomes included the presence of 1) at least one solid lung nodule (volume ≥30 mm^3^) and 2) a clinically relevant lung nodule (volume ≥100 mm^3^). Fully adjusted multivariable logistic regression models were applied overall and stratified by smoking status to identify independent risk factors for the presence of nodules.

**Results:**

Among the 12 055 participants (44.1% male; median age 60 years; 39.9% never-smokers; 98.7% White), we found lung nodules in 41.8% (5045 out of 12 055) and clinically relevant nodules in 11.4% (1377 out of 12 055); the corresponding figures among never-smokers were 38.8% and 9.5%, respectively. Factors independently associated with increased odds of having any lung nodule included male sex, older age, low educational level, former smoking, asbestos exposure and COPD. Among never-smokers, a family history of lung cancer increased the odds of both lung nodules and clinically relevant nodules. Among former and current smokers, low educational level was positively associated with lung nodules, whereas being overweight was negatively associated. Among current smokers, asbestos exposure and low physical activity were associated with clinically relevant nodules.

**Conclusions:**

The study provides a large-scale evaluation of lung nodules and associated risk factors in a Western European general population: lung nodules and clinically relevant nodules were prevalent, and never-smokers with a family history of lung cancer were a non-negligible group.

## Shareable abstract

**Lung nodules were prevalent in a Western European general population and risk factors associated with nodule presence may help to select the most suitable individuals/subgroups for screening and optimise screening eligibility criteria** https://bit.ly/3W0JBPy

## Graphical Abstract

**Figure:**
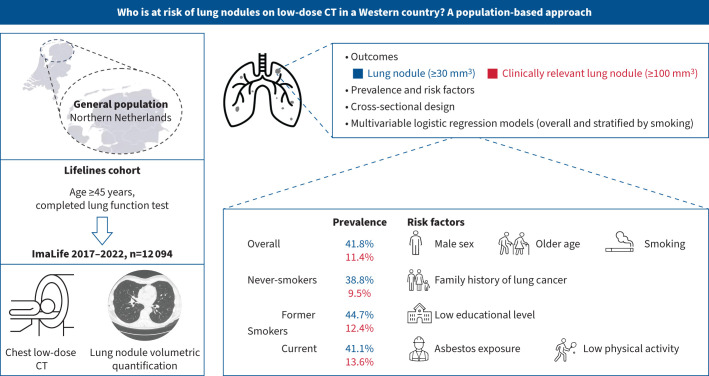
Overview of the risk factors associated with the presence of lung nodules in a Western European general population comprising current smokers, former smokers and never-smokers. CT: computed tomography.

## Introduction

Lung nodules are increasingly identified in asymptomatic individuals [1]. Although >95% of incidentally detected nodules on low-dose computed tomography (LDCT) are benign, they do have clinical importance as some may represent early and potentially curable malignancy [2]. Current guidelines emphasise a systematic approach to the evaluation of lung nodules, with risk stratification by individual and nodule characteristics [3]. Established characteristics include older age and heavy smoking, whereas other characteristics show variation across studies, limiting our ability to identify subgroups or individuals at elevated risk for lung nodules in the general population.

Multiple lung cancer screening trials have identified numerous risk factors associated with lung nodules [4]. However, the data come from high-risk populations composed of (former) heavy smokers. By contrast, to date there is only limited knowledge about nodules in the general population because non-smokers, who are presumed healthy, do not routinely undergo CT scans. This is of clinical importance if we consider that never-smokers comprise up to 75% of the global population [5] and that 15–25% of lung cancers develop in this group [6]. The proportion of lung cancers attributable to smoking has been decreasing worldwide [6], particularly in Western countries where rates of cigarette smoking have decreased significantly in recent years. As such, factors other than smoking are increasingly likely to influence the incidence of lung nodules and cancer in the general population. Improving our understanding of the potential effects of factors associated with lung nodules in the general, non-smoking population is important.

Several risk factors associated with lung nodules in the general population or among never-smokers, such as exposure to second-hand smoke, the use of solid fuels and indoor cooking, and air pollution, have been implicated or demonstrated in Asian general populations [7–9]. The inherent differences between Asian and Western populations, including smoking patterns, dietary and cooking habits, and the prevalence of mycobacterial diseases (*e.g.* tuberculosis), preclude generalisation. Currently, we lack knowledge in Western populations about the risk factors associated with lung nodules in never-smokers, and indeed how these differ from those in smokers. The present study aimed to identify potential risk factors for lung nodules in a Western European general population comprising current, former and never-smokers.

## Methods

### Study design and population

The Lifelines study, initiated in 2006, is a multidisciplinary, prospective, population-based cohort study examining the health and health-related behaviours of 10% of the population (n=167 729) living in the northern Netherlands [10]. It employs a broad range of investigative procedures in assessing the biomedical, sociodemographic, behavioural, physical and psychological factors which contribute to the health and disease of the general population, with a special focus on multimorbidity and complex genetics. This Lifelines cohort is predominantly White and is broadly representative of the general population in the northern part of the Netherlands [10, 11]. Lifelines questionnaire data have been collected at baseline (2007–2013), first-round follow-up (2011–2015), second-round assessment (2014–2017) and second-round follow-up (2016–2019). Measurement data have also been collected for the baseline and second-round assessment, but the third-round assessment is ongoing.

The current study was conducted within the framework of the ImaLife imaging study, which is connected to the second-round assessment [12]. Since August 2017, 12 094 participants from the Lifelines cohort were invited to the ImaLife substudy in which a chest LDCT and assessment of pulmonary nodules was done. Included were Lifelines participants aged ≥45 years who had had a pulmonary function test at the second-round assessment in Lifelines. Performance of this pulmonary function test was largely related to the availability of time slots for this test. Excluded were those who had not had a pulmonary function test and could not undergo an LDCT scan for prespecified reasons [12]. In addition, participants in the ImaLife study were excluded under the following conditions: 1) pregnant women and 2) individuals who had undergone chest CT scans within the past year. In participants with a recent respiratory infection within 3 weeks prior to the ImaLife assessment, scans were scheduled 3 months later. For the present analysis, participants were excluded in case of 1) lung opacity that could not be defined as lung nodules (*e.g.* lung masses >3 cm in diameter) or 2) missing values on key variables (*e.g.* smoking status). We defined a nodule as present if a participant had at least one solid lung nodule measuring ≥30 mm^3^ and defined nodules as absent if none were detected or the detected nodules measured <30 mm^3^, consistent with the ImaLife study protocol. The current analysis only considered solid lung nodules because subsolid nodules hold a distinct character from solid nodules, their volumetry measurement is not accurate and their management is different [3]. A lung nodule measuring ≥100 mm^3^ was defined as clinically relevant based on the increased probability of lung cancer [13]. Existing nodule management recommendations (supplementary table S6) also usually include a 100 mm^3^ volume/6 mm diameter cut-off value for follow-up or further management [3, 14]. Based on the ImaLife protocol, participants with intermediately sized nodules (100–300 mm^3^) were invited for a 3-month follow-up CT scan to study nodule persistence; participants with nodules sized ≥300 mm^3^ were referred to their general practitioner for further investigation, with the lung cancer diagnostic and outcome data being part of the third-round assessment of the Lifelines study. In participants with multiple lung nodules, only the largest nodule was selected for analysis.

All individuals gave their informed consent to participate in the Lifelines and ImaLife studies. The ImaLife study was approved by the Medical Ethics Committee of the University Medical Center Groningen, Groningen, the Netherlands (METc 2016-436).

### Chest LDCT acquisition and image analysis

All participants underwent chest LDCT using a third-generation dual-source CT scanner (SOMATOM Force; Siemens Healthineers, Erlangen, Germany). The CT scanning acquisition parameters are detailed elsewhere [12]. The presence or absence of solid lung nodules was determined by one of six trained radiologists (-in-training) (R. Vliegenthart, M. Rook, G. Kramer, Ahmed Aown, Marius G.J. Kok and J. Cai) with 4–15 years of experience, or by a well-trained technical physician (G.J. Pelgrim) under the supervision of an experienced radiologist. Readers measured the volume of each solid lung nodule by using syngo.via software with the MM Oncology application version VB30 (Siemens Healthineers). If inappropriate automated measurements occurred, especially for irregularly shaped and vascular-attached lung nodules, the readers could manually adjust and modify the measurement.

### Data collection

For all participants, we extracted the following data from self-reported questionnaires and measurements in Lifelines: sociodemographic data (*i.e.* age at CT scan, ethnicity, sex and educational level), medical history (*i.e.* cardiovascular disease, COPD, diabetes, family history of lung cancer, allergies (dust, pollen, animal) and overweight/obesity), smoking exposure (*i.e.* smoking status (never, former, current), cumulative smoking intensity (pack-years), age at smoking initiation and years since quitting smoking), second-hand smoke exposure, occupation exposure (*i.e.* asbestos exposure) and lifestyle (*i.e.* total physical activity and alcohol intake). The current analysis included data from the second-round assessment in Lifelines (2014–2017), which was closest to the time of LDCT scanning (2017–2022). In case of missing values at the second-round assessment, we used data collected at the baseline assessment (2007–2013) and the first-round follow-up (2011–2015). For the definitions of all potential risk factors, see supplementary table S1.

### Statistical analysis

Numerical variables are presented as median with interquartile range (IQR) and categorical data are presented as absolute number with percentage. Multivariable logistic regression analyses were performed to identify independent risk factors for lung nodules and clinically relevant lung nodules among sociodemographic factors, medical history, smoking exposure, occupational exposure and lifestyle variables. Odds ratios and 95% confidence intervals were reported. Analyses were performed for the total population and stratified by smoking status. All tests were two-sided and considered statistically significant for p-values <0.05. Statistical analyses were performed using SPSS version 28.0 (IBM, Armonk, NY, USA).

## Results

### Population characteristics

**FIGURE 1:**
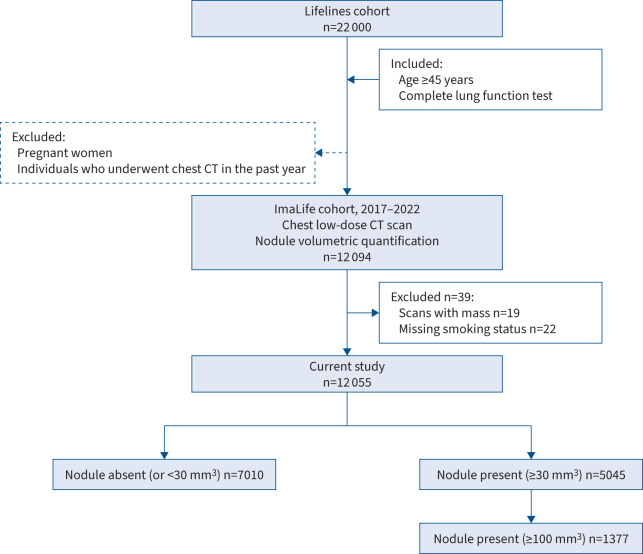
Study flowchart. Individuals could have more than one reason for exclusion. CT: computed tomography.

After excluding 19 individuals with pulmonary masses and 22 with no information on their smoking status, we finally included 12 055 participants (median (IQR) age at CT scan 60.1 (53.4–69.7) years; 44.1% male; 98.7% White) (figure 1). In the ImaLife population, 39.9% were never-smokers, 46.6% were former smokers (median (IQR) 7.9 (3.3–15.0) pack-years) and 13.5% were current smokers (median (IQR) 17.7 (10.2–26.60) pack-years). Former smokers had quit a median (IQR) of 27.4 (16.8–38.7) years earlier (table 1). Among the overall population, 5045 (41.8%) participants, comprising 2502 males and 2543 females with a median (IQR) age of 61.2 (55.2–72.8) years, had at least one lung nodule ≥30 mm^3^. Of these, 1868 (38.8%) were never-smokers, 2511 (44.7%) were former smokers and 666 (41.1%) were current smokers. Multiple lung nodules (≥30 mm^3^) were present in 21.0% of current smokers and 21.0% of former smokers, both of which were higher than the 15.9% of never-smokers. Clinically relevant nodules (≥100 mm^3^) were identified in 1377 participants (11.4% overall) with a median (IQR) age of 65.3 (57.3–75.2) years and a slight male preponderance (54.8% male). By smoking status, they were present in 9.5% of never-smokers, 12.4% of former smokers and 13.6% of current smokers. Participants with lung nodules or clinically relevant nodules were more likely to be older than those without nodules, regardless of smoking status. Among both the former and current smokers, smoking intensities were higher in the groups with nodules than in those without nodules (table 2).

**TABLE 1:** Baseline characteristics of the population overall and stratified by smoking status

	Overall	Smoking status		
		Never-smoker	Former smoker	Current smoker
**Participants**	12 055 (100)	4813 (39.9)	5620 (46.6)	1622 (13.5)
**Sex**				
Female	6743 (55.9)	2847 (59.2)	3063 (54.5)	833 (51.4)
Male	5312 (44.1)	1966 (40.8)	2557 (45.5)	789 (48.6)
**Age, years**	60.1 (53.4–69.6)	57.9 (51.5–67.5)	62.3 (56.4–72.5)	57.2 (51.3–61.8)
**Age at CT scan**				
45–55 years	4106 (34.1)	2077 (43.2)	1322 (23.5)	707 (43.6)
56–65 years	3972 (32.9)	1401 (29.1)	1953 (34.8)	618 (38.1)
≥66 years	3977 (33.0)	1335 (27.7)	2345 (41.7)	297 (18.3)
**Ethnicity**				
White	11 275 (98.7)	4495 (99.1)	5288 (98.8)	1492 (98.0)
Non-White^#^	140 (1.3)	45 (0.9)	64 (1.2)	31 (2.0)
**Educational level**				
High	3556 (30.2)	1601 (34.2)	1575 (28.7)	380 (23.8)
Moderate	6137 (52.1)	2398 (51.2)	2880 (52.4)	859 (53.8)
Low	2086 (17.7)	687 (14.7)	1042 (19.0)	357 (22.4)
**Pack-years** ^¶^	9.5 (4.0–18.5)		7.9 (3.3–15.0)	17.7 (10.2–26.6)
**Age of starting smoking** ^¶^				
>18 years	1218 (16.9)		913 (16.3)	305 (18.9)
≤18 years	5999 (83.1)		4692 (83.7)	1307 (81.1)
**Years since quitting** **^+^**				
>15 years	4432 (79.0)		4432 (79.0)	
≤15 years	1176 (21.0)		1176 (21.0)	
**Years since quitting** **^+^**	27.4 (16.8–38.7)		27.4 (16.8–38.7)	
**Second-hand smoke exposure**				
No	8490 (72.0)	3660 (78.1)	4105 (74.6)	725 (45.4)
Yes	3295 (28.0)	1026 (21.9)	1396 (25.4)	873 (54.6)
**Alcohol intake**				
None/mild (0–1 drink-days·week^−1^)	5158 (47.5)	2536 (58.0)	1996 (40.3)	626 (40.7)
Moderate (2–3 drink-days·week^−1^)	2758 (25.4)	1040 (23.8)	1344 (27.1)	374 (24.3)
Heavy (≥4 drink-days·week^−1^)	2943 (27.1)	793 (18.2)	1611 (32.5)	539 (35.0)
**Asbestos exposure**				
No	11 006 (94.4)	4391 (94.6)	5125 (94.3)	1490 (94.5)
Yes	649 (5.6)	251 (5.4)	312 (5.7)	86 (5.5)
**BMI**				
Normal (<25.0 kg·m^−2^)	4875 (40.4)	2148 (44.6)	2033 (36.2)	694 (42.8)
Obese/overweight (≥25.0 kg·m^−2^)	7179 (59.6)	2665 (55.4)	3586 (63.8)	928 (57.2)
**Physical activity**				
High (≥5 days·week^−1^ 30 min activity)	5657 (52.2)	2270 (52.8)	2739 (54.1)	648 (44.3)
Low (1–4 days·week^−1^ 30 min activity)	5170 (47.8)	2030 (47.2)	2326 (45.9)	814 (55.7)
**Family history of lung cancer**				
No	10 460 (90.8)	4198 (91.6)	4858 (90.0)	1404 (91.2)
Yes	1059 (9.2)	385 (8.4)	539 (10.0)	135 (8.8)
**Cardiovascular disease**				
No	11 727 (97.5)	4707 (98.1)	5434 (96.9)	1586 (98.1)
Yes	296 (2.7)	93 (1.9)	172 (3.1)	31 (1.9)
**Diabetes**				
No	11 802 (97.9)	4740 (98.5)	5460 (97.2)	1602 (98.9)
Yes	250 (2.1)	73 (1.5)	159 (2.8)	18 (1.1)
**COPD**				
No	9258 (78.2)	3997 (84.3)	4154 (75.8)	1107 (68.8)
Yes	2577 (21.8)	747 (15.7)	1327 (24.2)	503 (31.2)
**Allergy**				
No	9199 (76.4)	3519 (73.3)	4380 (78.0)	1300 (80.2)
Yes	2837 (23.6)	1285 (26.7)	1232 (22.0)	320 (19.8)
**Lung nodule (≥30 mm^3^)**				
Nodule absence	7010 (58.2)	2945 (61.2)	3109 (55.3)	956 (58.9)
Solitary	2755 (22.9)	1101 (22.9)	1329 (23.6)	325 (20.0)
Multiple	2290 (19.0)	767 (15.9)	1182 (21.0)	341 (21.0)

Data are presented as n (%) or median (interquartile range). CT: computed tomography; BMI: body mass index. ^#^: non-White includes Asian, African and others; ^¶^: smokers only; ^+^: quitters only. Missing values: ethnicity, 5.3%; educational level, 2.3%; pack-years, 6.0%; age of starting smoking, 0.2%; years since quitting, 0.1%; second-hand exposure, 2.2%; alcohol intake, 9.9%; asbestos exposure, 3.3%; physical activity, 10.2%; family history of lung cancer, 4.4%; cardiovascular disease, 0.3%; COPD, 1.8%; allergy, 0.2%.

**TABLE 2:** Characteristics of participants for the presence or absence of lung nodules in the Western European general population stratified by smoking status

	Overall (n=12 055)		Never-smoker (n=4813)		Former smoker (n=5620)		Current smoker (n=1622)	
	Present	Absent	Present	Absent	Present	Absent	Present	Absent
**Nodules (≥30 mm^3^)**								
Sex								
Female	2543 (50.4)	4200 (59.9)	1018 (54.5)	1829 (62.1)	1212 (48.3)	1851 (59.5)	313 (47.0)	520 (54.4)
Male	2502 (49.6)	2810 (40.1)	850 (45.5)	1116 (37.9)	1299 (51.7)	1258 (40.5)	353 (53.0)	436 (45.6)
Age, years	61.2 (55.2–72.8)	58.8 (52.3–66.9)	60.3 (53.1–71.6)	56.7 (50.7–64.3)	65.8 (58.2–75.2)	60.6 (55.3–69.9)	58.5 (53.1–65.0)	56.6 (50.4–61.0)
Age at CT scan								
45–55 years	1393 (27.6)	2713 (38.7)	677 (36.2)	1400 (47.5)	467 (18.6)	855 (27.5)	249 (37.4)	458 (47.9)
56–65 years	1594 (31.6)	2378 (33.9)	533 (28.5)	868 (29.5)	799 (31.8)	1154 (37.1)	262 (39.3)	356 (37.2)
≥66 years	2058 (40.8)	1919 (27.4)	658 (35.2)	677 (23.0)	1245 (49.6)	1100 (35.4)	155 (23.3)	142 (14.9)
Pack-years^#^	10.5 (4.5–20.0)	9.0 (3.8–17.1)			8.5 (3.8–17.0)	7.3 (3.0–14.0)	20.0 (11.5–29.0)	16.5 (9.4–24.6)
Years since quitting^¶^	28.6 (17.3–38.9)	26.3 (16.2–37.1)			28.6 (17.3–38.9)	26.3 (16.2–37.1)		
**Clinically relevant nodules (≥100 mm^3^)**								
Sex								
Female	623 (45.2)	6120 (57.3)	214 (46.8)	2633 (60.4)	311 (44.5)	2752 (55.9)	98 (44.3)	735 (52.5)
Male	754 (54.8)	4558 (42.7)	243 (53.2)	1723 (39.6)	388 (55.5)	2169 (44.1)	123 (55.7)	666 (47.5)
Age, years	65.3 (57.3–75.2)	59.7 (53.1–68.8)	62.1 (55.3–75.2)	57.4 (51.3–66.4)	68.6 (60.3–76.3)	61.2 (56.0–71.6)	59.3 (53.6–66.1)	44.4 (51.0–61.3)
Age at CT scan								
45–55 years	297 (21.6)	3809 (35.7)	130 (28.4)	1947 (44.7)	92 (13.2)	1230 (25.0)	75 (33.9)	632 (45.1)
56–65 years	415 (30.1)	3557 (33.3)	125 (27.4)	1276 (29.3)	201 (28.8)	1752 (35.6)	89 (40.3)	529 (37.8)
≥66 years	665 (48.3)	3312 (31.0)	202 (44.2)	1133 (26.0)	406 (58.1)	1939 (39.4)	57 (25.8)	240 (17.1)
Pack-years^#^	11.3 (5.0–21.4)	9.3 (4.0–18.0)			9.3 (4.3–18.5)	7.5 (3.3–14.8)	20.1 (10.3–29.3)	17.5 (10.2–26.3)
Years since quitting^¶^	30.5 (18.5–41.2)	27.1 (16.5–37.5)			30.5 (18.5–41.2)	27.1 (16.5–37.5)		

Data are presented as n (%) or median (interquartile range). CT: computed tomography. ^#^: smokers only; ^¶^: quitters only. Missing values: pack-years, 6.0%; years since quitting, 0.1%.

### Risk factors associated with lung nodules

**FIGURE 2:**
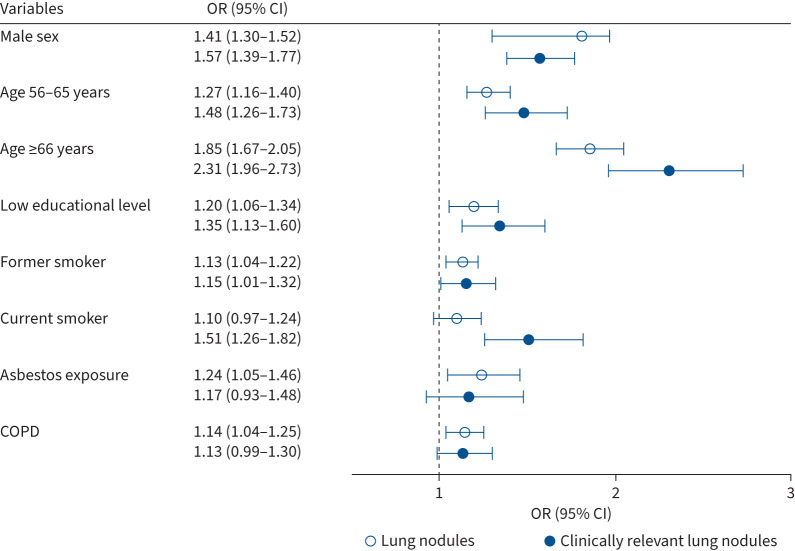
Odds ratios for risk factors independently associated with at least one lung nodule in the general population. We differentiated individuals into those with solid lung nodules (≥30 mm^3^) or clinically relevant lung nodules (≥100 mm^3^).

In the overall general population, multivariable regression analysis revealed independent associations with an increased odds of lung nodule presence for male sex (OR 1.41, 95% CI 1.30–1.52), age 56–65 years (OR 1.27, 95% CI 1.16–1.40), age ≥66 years (OR 1.85, 95% CI 1.67–2.05), low educational level (OR 1.20, 95% CI 1.06–1.34), former smokers (OR 1.13, 95% CI 1.04–1.22), asbestos exposure (OR 1.24, 95% CI 1.05–1.46) and COPD (OR 1.14, 95% CI 1.04–1.25). Factors associated with an increased odds of clinically relevant lung nodules included male sex (OR 1.57, 95% CI 1.39–1.77), age 56–65 years (OR 1.48, 95% CI 1.26–1.73), age ≥66 years (OR 2.31, 95% CI 1.96–2.73), low educational level (OR 1.35, 95% CI 1.13–1.60) and former smokers (OR 1.15, 95% CI 1.01–1.32), but also included current smokers (OR 1.51, 95% CI 1.26–1.82). Overweight/obesity (OR 0.87, 95% CI 0.77–0.98) was associated with a decreased odd of having clinically relevant nodules (figure 2 and supplementary table S2).

### Risk factors associated with lung nodules by smoking status

**FIGURE 3:**
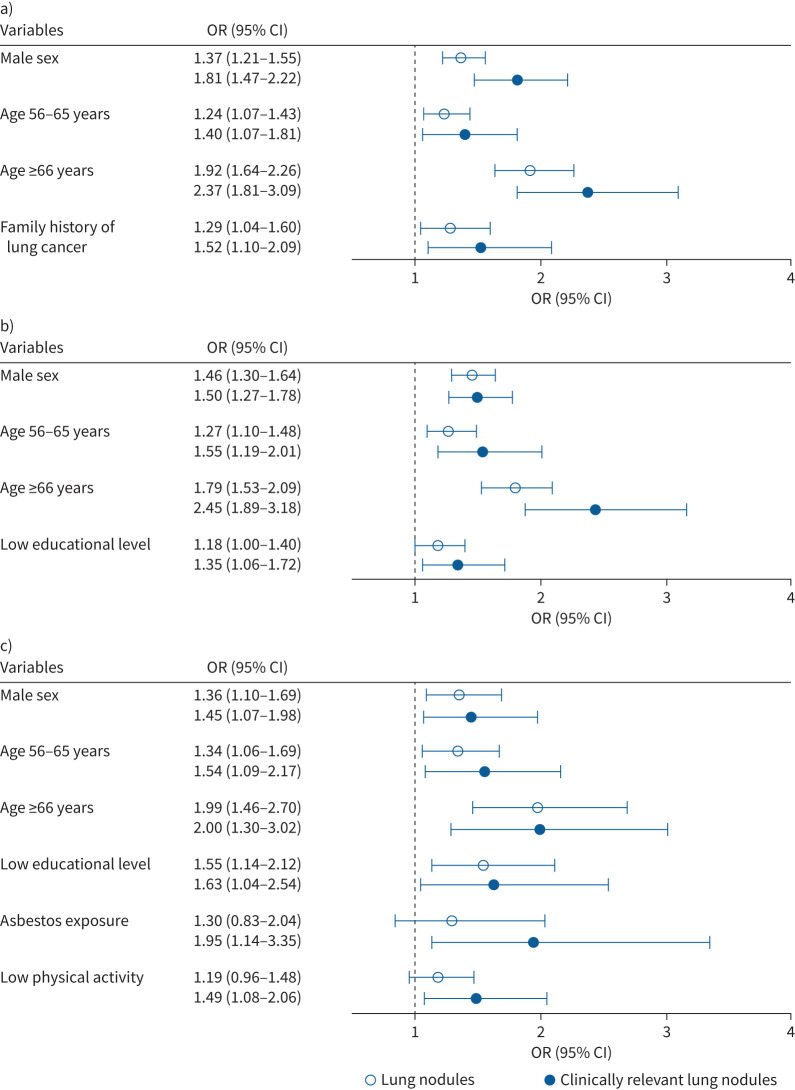
Odds ratios of risk factors independently associated with at least one lung nodule by smoking status. We differentiated individuals into those with solid lung nodules (≥30 mm^3^) or clinically relevant lung nodules (≥100 mm^3^) by subgroups of a) never-smokers, b) former smokers and c) current smokers.

Older age and male sex remained significantly associated with an increased odds of lung nodules, irrespective of smoking status. Notably, only never-smokers exhibited significant associations for lung nodules (OR 1.29, 95% CI 1.04–1.60) and clinically relevant lung nodules (OR 1.52, 95% CI 1.10–2.09) with a family history of lung cancer (figure 3a and supplementary table S3). Low educational level was significantly and positively associated with lung nodules in former (OR 1.18, 95% CI 1.00–1.40) and current (OR 1.55, 95% CI 1.14–2.12) smokers, and with clinically relevant lung nodules in former (OR 1.35, 95% CI 1.06–1.72) and current (OR 1.63, 95% CI 1.04–2.54) smokers (figure 3b and c and supplementary tables S4 and S5). Asbestos exposure (OR 1.95, 95% CI 1.14–3.35) and low physical activity (OR 1.49, 95% CI 1.08–2.06) were associated with an increased risk of having clinically relevant lung nodules in current smokers (figure 3c and supplementary table S5). Finally, overweight/obesity was negatively associated with the presence of clinically relevant lung nodules in former (OR 0.83, 95% CI 0.70–0.99) and current (OR 0.71, 95% CI 0.52–0.97) smokers.

## Discussion

In this large population-based study, we evaluated a range of potential risk factors associated with lung nodules in a Western European general population comprising mostly non-smokers. Lung nodules were present in 41.8% of the overall population and in 38.8% of never-smokers. We demonstrated that male sex, older age, low educational level, smoking, asbestos exposure and COPD independently increased the probability of lung nodules in the overall population. Comparable results were found for risk factors associated with clinically relevant lung nodules (prevalence 11.4%). Notably, a family history of lung cancer was significantly and positively associated with lung nodules among never-smokers. By contrast, among former and current smokers, low educational level was associated with an increased probability of having either lung nodules or clinically relevant nodules, whereas being overweight/obesity was associated with a decreased probability of having clinically relevant nodules. Asbestos exposure and low physical activity were only associated with clinically relevant lung nodules in current smokers.

The relationship between age and lung nodule presence has been observed worldwide in both lung cancer screening studies (thus high-risk populations) and general populations [1, 3, 15, 16]. Consistent with previous studies, older age groups were more likely to have lung nodules in our study, with the probability among those aged ≥66 years being more than double that of people aged 45–55 years. The odds ratio increased with age, and this trend remained irrespective of smoking status. Despite being a non-modifiable risk factor, age therefore contributes to the presence of lung nodules in Western European general populations. This may result from longer cumulative durations of environmental exposure, (indolent) infections and inflammation with or without granuloma development, and reactive lymph node enlargement. Furthermore, the other risk factors in this study were also age-dependent to varying degrees [1].

Male sex has been considered an important risk factor for lung nodule development in Western populations, with the most widely accepted interpretation being that this reflects differences in smoking behaviours between men and women [17]. The relationship between smoking and lung nodule risk is well known to be dose-dependent, with risk increasing as the duration and amount of active smoking increases [18, 19]. Male and female smokers had medians of 12.0 and 7.8 pack-years in this study, respectively. Apart from the inherent gender difference in genetic susceptibility, the greater smoking volume among males may partly explain the observed differences. According to previous studies, other risk factors unrelated to smoking (*e.g.* lifestyle or occupational exposure) could also account for some of the sex differences [20].

A strong causal association exists between smoking and lung nodule development [21]. Moreover, smoking is of particular interest because it can be modified to improve health. In recent years, the percentage of smokers among both men and women in the Netherlands has declined [22]; however, the incidence of lung cancer is still rising [23]. Compared with never-smokers, those who smoke have an approximately 10–20-fold increased risk of developing a malignant lung nodule [24]. A study of lung cancer screening showed that almost 50% of all smokers aged >50 years had at least one nodule, with 10% developing a new nodule in the subsequent year [25]. Consistent with the existing body of knowledge, we observed that current smoking was associated with a significantly increased risk of clinically relevant nodules (OR 1.51, 95% CI 1.26–1.82). Although this did not apply to former or never-smokers, the relatively small proportion of current smokers (13.5%) and the possibility of smoking having less pronounced effects at the population level necessitate care when interpreting our findings among smokers. In addition, we observed a significant positive association between clinically relevant nodule presence and asbestos exposure among current smokers. Asbestos exposure has been attributed as the most important occupational risk factor for the development of both lung nodules and lung cancer, with previous studies showing that cumulative exposure and synergism between asbestos and tobacco smoke has a pathophysiological role in malignant lung nodule development [26]. Given the relatively small subgroup exposed to asbestos in the overall population (5.4%), future research should use quantitative assessments of asbestos exposure intensity instead of binary outcomes.

Former smoking was also associated with an increased risk of lung nodules and clinically relevant nodules. The predominance of former smokers among smokers (46.6% former *versus* 13.5% current) in our study is in line with the current Dutch adult population (2022: 32.8% former *versus* 18.9% current) [22]. The former smokers in our study had relatively lower smoking intensities (median 7.9 pack-years), much lower than in screening populations (*e.g.* NELSON study: median (IQR) 38.0 (29.7–49.5) pack-years [27]), and they had relatively longer periods of smoking cessation (median 27.4 years). This indicated that smoking potentially had a relatively lower impact in our included former smokers. However, the risk of former smokers having a nodule remained elevated relative to never-smokers. A previous study also revealed that the risk of lung cancer in former smokers remains three-fold higher than in never-smokers, even 25 years after quitting, which is beyond the current window for screening eligibility [28]. Furthermore, the Netherlands has been at the forefront of tobacco control in recent years compared to other Western European countries, and the proportion of former smokers among Dutch adults is increasingly dominant [22]. Therefore, it remains critical that we identify risk factors associated with lung nodule development in former smokers.

Previous studies have shown that a family history of lung cancer, as a representative genetic factor, played a role in predisposing individuals to lung cancer [29, 30]. For instance, a family history of lung cancer among first-degree relatives is associated with a 50% higher risk of developing malignant lung nodules [30]. Given the comparable prevalence of nodules among never-smokers and smokers in the present study, factors other than smoking must have contributed to the presence of lung nodules. Indeed, a family history of lung cancer was only pronounced as a risk factor among never-smokers with either lung nodules or clinically relevant nodules. This may reflect the hereditary nature of genetic susceptibility for the development of lung nodules or a familial aggregation of malignant lung nodules due to shared lifestyle and environmental factors. This positive significant association remained despite performing a fully adjusted regression analysis among never-smokers to minimise the effects of smoking and related confounders (*e.g.* second-hand smoke). This has implications for the implementation of lung cancer screening in high-risk individuals who have never smoked, because current screening eligibility criteria only target heavy smokers, and as such, could miss a considerable number of lung cancers in never-smokers. However, it is not cost-effective to screen all never-smokers. Identifying and screening never-smokers with sufficient risk would be a more cost-effective approach, but this will require the development of accurate risk assessment tools (*e.g.* including family history of lung cancer) to identify high-risk never-smokers.

Educational level served as a proxy for socioeconomic status in our study, and a low educational level was associated with an increased risk of having lung nodules and clinically relevant nodules overall and among former and current smokers. A lower socioeconomic status might increase susceptibility to chronic diseases through exposure to other environmental stressors (*e.g.* poor housing conditions and occupational exposures), unhealthy lifestyles or less inclination to seek medical care. Subsequently, these factors may lead to the development of relatively more lung nodules compared with higher socioeconomic status. Finally, obese/overweight was associated with a reduced probability of having clinically relevant nodules, which was observed in the general population and in former and current smokers. We are currently unable to explain this result and have found no direct correlation between obesity/overweight and a reduced probability of having nodules. Although some indirect evidence exists, such as the role of dietary factors or daily exercise, these also present conflicting results [31].

Previous studies indicated that a number of prior intrapulmonary diseases are associated with lung nodule presence, but this issue could not be investigated in our study. Participants were not asked about these types of diseases in the Lifelines study. Furthermore, these diseases are not common in the Netherlands (*e.g.* estimated prevalence of sarcoidosis: 20 cases per 100 000 inhabitants (https://sarcoidose.nl)). Incidence of mycobacterium infection and tuberculosis in the Netherlands (around 2.3–4.5 per 100 000 inhabitants each [32–34]) is very low, and the latter occurs mainly among foreign-born immigrants [33]. Thus, we expect limited impact on our results.

To the best of our knowledge, no other population-based imaging study has investigated this topic with a large sample size and sufficient statistical power for effect size estimations and subgroup analyses. This study is derived from the ImaLife substudy of the Lifelines cohort, which is so far the only general population-based LDCT scanning study that includes a large cohort of never-smokers. Other studies that have provided lung nodule data using LDCT scanning had prespecified risk factors, and inclusion/exclusion criteria, and almost all of them excluded subjects who had never smoked. Therefore, we believe our study provides unique data on the overall prevalence of lung nodules and clinically relevant nodules and risk factors associated with their presence, particularly in never-smokers. Furthermore, the Lifelines cohort has been validated, and shown to have a low risk of selection bias and to be generalisable to the population in the north of the Netherlands [11].

Nevertheless, several limitations warrant consideration. First, this study was at risk of information bias because most population characteristics were based on self-reported questionnaires. Although this bias could never be prevented entirely and could have led to misclassification, we think that the large number of subjects prevented this misreporting from materially affecting the interpretation of our results. Second, despite identifying several significant risk factors, the cause–effect relationship with the presence of lung nodules cannot be established owing to the cross-sectional design. Third, current smokers who may have quit smoking during the study period were not considered, and we instead assumed that smoking behaviours were persistent throughout. However, 95.8% of the data on smoking status came from the Lifelines second-round assessment and follow-up, closest in time to the ImaLife study. So, we believe this had a limited impact on our final conclusions. Furthermore, for some medical/lifestyle risk factors (*e.g.* diabetes, cardiovascular disease, COPD and number of years smoking) that may affect nodule presence, data at the second-round examination were missing, and data from the first round were used. Considering that this group of participants represents only a small percentage (4.0–8.1%), these changes did not materially affect the interpretation of the presented results. Fourth, all participants in our study were from the northern part of the Netherlands, almost exclusively of Northern/Western European descent (98.7%), indicating that this is a relatively homogenous population. Our results may not be generalisable to populations with other racial distributions. Fifth, participants with actionable findings were referred to their primary care physician for further management and diagnosis. We do not have the lung cancer diagnosis and outcome data at this moment; this will be available in the Lifelines study in the future.

In conclusion, male sex, older age, low educational level, smoking, asbestos exposure and COPD were associated with the presence of lung nodules in this Western European general population (39.9% never-smokers). We also provide evidence that a family history of lung cancer contributes to the presence of lung nodules in never-smokers. These risk factors could be used to identify individuals or subgroups at elevated risk of clinically suspicious lung nodules and may help to optimise the eligibility criteria for lung cancer screening in Western countries.

## Supplementary material

10.1183/13993003.01736-2023.Supp1 **Please note:** supplementary material is not edited by the Editorial Office, and is uploaded as it has been supplied by the author.
Supplementary material ERJ-01736-2023.Supplement

## Shareable PDF

10.1183/13993003.01736-2023.Shareable1 This one-page PDF can be shared freely online.
Shareable PDF ERJ-01736-2023.Shareable

## Data Availability

The data that support the findings of the present study are available through Lifelines (www.lifelines.nl); however, data access is restricted. Requests for data access can be directed to the Lifelines Cohort Study and Biobank (www.lifelines.nl/researcher/how-to-apply).
