# Supplementary material for: Who is at risk of lung nodules on low-dose CT in a Western country? A population-based approach
Source: Eur Respir J. 2024 Jun 6;63(6):2301736. doi: 10.1183/13993003.01736-2023 (PMC11154756; doi:10.1183/13993003.01736-2023)
Supplement: Supplementary file 1 [file ERJ-01736-2023.Supplement.pdf]

Supplementary materials

Supplementary Table S1: variable assignment

**Variable**	**Definition**	**Assignment**
Outcome variables		
Group-1	Participants without any lung nodules, or with nodule volume of < 30 mm^3	1: nodule absent
	Participants with at least one solid lung nodule with a volume of ≥30 mm^3	2: nodule present
Group-2	Participants without any lung nodules, or with nodule volume of < 100 mm^3	1: clinically relevant nodule absent
	Participants with at least one solid lung nodule with a volume of ≥100 mm^3	2: clinically relevant nodule present
Potential risk factors		
Age at CT scan	Age of participants for chest low-dose CT scan	1: 45-55 years
		2: 55-65 years
		3: ≥65 years
Ethnicity	Determined based on the question: “Which of the following population do you consider yourself belong to?”	1: White
		2: Non-white
Sex	Sex of the participants	1: male
		2: female
Educational level	Highest level of education attained derived using the International Standard Classification of Education (ISCED)	1: high level<br>2: moderate level<br>3: low level
Smoking status	Never smokers were defined as those who answered ’no’ to the question “Have you ever smoked for as long as a year?”. Former smokers were defined as those who reported being smokers for more than a year but had stopped smoking for at least 1 month at the time of the questionnaire. Current smokers were defined as those who answered ‘yes’ to the question “Do you smoke now or have you	0: never smoker<br>1: former smoker<br>2: current smoker

	been smoking in the last month?”.	
Pack-years	Calculated by multiplying intensity in packs/day by duration in years with one pack containing 20 cigarettes per day, with a threshold of 20 as light/moderate and heavy.	
Age of starting smoking	Determined based on the question: “How old were you when you started smoking?”	0: >18 years<br>1: ≤18 years
Years since quitting	Calculated from the question: “If you no longer smoke, how old were you when you stopped smoking?”	0: >15 years<br>1: ≤15 years
SHS exposure	Participant’s passive smoking exposure at home or at work, or both	0: no; 1: yes
Physical activity	Calculated by multiplying the number of days with 30 min physical activity per week (e.g., walking, household activities, sports, and gardening)	0: high (≥5 days/wk 30 min activity)<br>1: low (0–4 days/wk 30 min activity)
Alcohol intake	Defined as the total number of days have an alcohol drink in a week	0: no drinking/mild (0-1 drink-day /wk)<br>1: moderate (1–3 drink-days /wk)<br>2: heavy (≥4 drink-days/wk)
BMI	Measured weight (kg) divided by measured height square (m^2)	1: normal (<25.0 kg/m^2)<br>2: overweight/obesity (≥ 25.0 kg/m^2)
Asbestos exposure	Defined based on the International Standard Classification of Occupations – ISCO-08	0: no; 1: yes
Family history of lung cancer	Defined as self-reported family history of lung cancer	0: no; 1: yes
Diabetes	Defined as self-reported history of diabetes/mellitus	0: no; 1: yes
Allergy	Defined as self-reported history of allergies to dust/pollen/animal	0: no; 1: yes
Cardiovascular disease	Defined as self-reported history of myocardial infarction or stroke or heart failure	0: no; 1: yes
COPD	Defined as the FEVl/FVC less than 0.70 based on the pulmonary function test at 2nd assessment	0: no; 1: yes

Abbreviations: SHS exposure, secondhand smoke exposure; BMI, body mass index; COPD, chronic obstructive pulmonary disease; FEV_1, forced expiratory volume in 1 second; FVC, Forced vital capacity.

Supplementary Table S2: Multivariable logistic regression analysis of the relationship between population characteristics and lung nodules in the general population (N = 12055)

	Lung nodules (volume ≥ 30mm^3)	Clinically relevant lung nodules (volume ≥ 100mm^3)
	**OR [95%CI]**	**OR [95%CI]**
**Sex**		
Female	Ref	Ref
Male	**1.41 [1.30-1.52] ***	**1.57 [1.39-1.77] ***
**Age at CT scan, years**		
45-55	Ref	Ref
56-65	**1.27 [1.16-1.40] ***	**1.48 [1.26-1.73]****
≥66	**1.85 [1.67-2.05] ***	**2.31 [1.96-2.73] ***
**Education level**		
High	Ref	Ref
Moderate	1.02 [0.94-1.12]	1.13 [0.98-1.30]
Low	**1.20 [1.06-1.34] ****	**1.35 [1.13-1.60]***
**Smoking status**		
Never smoker	Ref	Ref
Former smoker	**1.13 [1.04-1.22] ****	**1.15 [1.01-1.32]****
Current smoker	1.10 [0.97-1.24]	**1.51 [1.26-1.82]***
**SHS exposure**		
No	Ref	Ref
Yes	0.99 [0.91-1.09]	0.98 [0.86-1.14]
**Alcohol use**		
No drinking/mild	Ref	Ref
Moderate	0.95 [0.87-1.05]	0.89 [0.76-1.04]
Heavy	1.02 [0.92-1.12]	0.98 [0.85-1.14]
**Asbestos exposure**		
No	Ref	Ref
Yes	**1.24 [1.05-1.46] ****	1.17 [0.93-1.48]
**Body Mass Index**		
Normal	Ref	Ref
Overweight/obesity	0.95 [0.88-1.03]	**0.87 [0.77-0.98]****
**Physical activity**		
High	Ref	Ref
Low	0.99 [0.91-1.07]	1.12 [0.99-1.26]
**Family history of lung cancer**		
No	Ref	Ref
Yes	1.08 [0.95-1.23]	1.10 [0.90-1.33]
**Cardiovascular disease**		
No	Ref	Ref
Yes	1.02 [0.80-1.29]	0.82 [0.58-1.17]
**Diabetes**		

No	Ref	Ref
Yes	0.94 [0.72-1.21]	0.77 [0.52-1.14]
**COPD**		
No	Ref	Ref
Yes	**1.14 [1.04-1.25]** **	1.13 [0.99-1.30]
**Allergy**		
No	Ref	Ref
Yes	1.05 [0.96-1.15]	0.98 [0.85-1.13]

ORs in overall were adjusted by age, sex, educational level, smoking status, SHS exposure, alcohol use, asbestos exposure, Body Mass Index, physical activity, family history of lung cancer, cardiovascular disease, diabetes, COPD, allergy.

Abbreviations: SHS, secondhand smoke; COPD, chronic obstructive pulmonary disease; OR, odds ratio; 95%CI, 95% Confidence Interval.

*P value <0.001, ** P values<0.05

Supplementary Table S3: Multivariable logistic regression analysis of the relationship between population characteristics and lung nodules in never smokers (N = 4813)

	**Lung nodules (volume ≥ 30mm³)**	**Clinically relevant lung nodules (volume ≥ 100mm³)**
	**OR [95%CI]**	**OR [95%CI]**
**Sex**		
Female	Ref	Ref
Male	**1.37 [1.21-1.55] ***	**1.81 [1.47-2.22] ***
**Age at CT scan, years**		
45-55	Ref	Ref
56-65	**1.24 [1.07-1.43] *****	**1.40 [1.07-1.81]*****
≥66	**1.92 [1.64-2.26] ***	**2.37 [1.81-3.09] ***
**Education level**		
High	Ref	Ref
Moderate	0.94 [0.82-1.08]	1.03 [0.81-1.30]
Low	1.09 [0.89-1.33]	1.19 [0.87-1.62]
**SHS exposure**		
No	Ref	Ref
Yes	0.97 [0.83-1.13]	1.07 [0.83-1.39]
**Alcohol use**		
No drinking/mild	Ref	Ref
Moderate	0.96 [0.83-1.12]	0.79 [0.60-1.04]
Heavy	0.98 [0.83-1.16]	1.00 [0.75-1.32]
**Asbestos exposure**		
No	Ref	Ref
Yes	1.28 [0.98-1.67]	1.08 [0.71-1.64]
**Body Mass Index**		
Normal	Ref	Ref
Overweight/obesity	0.99 [0.87-1.12]	1.00 [0.82-1.23]
**Physical activity**		
High	Ref	Ref
Low	0.92 [0.81-1.04]	1.16 [0.93-1.44]
**Family history of lung cancer**		
No	Ref	Ref
Yes	**1.29 [1.04-1.60]*****	**1.52 [1.10-2.09]*****
**Cardiovascular disease**		
No	Ref	Ref
Yes	1.00 [0.65-1.53]	0.84 [0.42-1.67]
**Diabetes**		
No	Ref	Ref
Yes	0.75 [0.46-1.23]	0.49 [0.19-1.25]
**COPD**		
No	Ref	Ref

Yes	1.13 [0.96-1.33]	1.22 [0.95-1.58]
**Allergy**		
No	Ref	Ref
Yes	1.06 [0.93-1.21]	0.97 [0.77-1.22]

ORs in never smoker were adjusted by age, sex, educational level, SHS exposure, alcohol use, asbestos exposure, Body Mass Index, physical activity, family history of lung cancer, cardiovascular disease, diabetes, COPD, allergy.

Abbreviations: SHS, secondhand smoke; OR, odds ratio; 95%CI, 95% Confidence Interval.

*P value <0.001, ** P values<0.05

Supplementary Table S4: Multivariable logistic regression analysis of the relationship between population characteristics and lung nodules in former smokers (N = 5620)

	Lung nodules (volume ≥ 30mm³)	Clinically relevant lung nodules (volume ≥ 100mm³)
	**OR [95%CI]**	**OR [95%CI]**
**Sex**		
Female	Ref	Ref
Male	**1.46 [1.30-1.64] ***	**1.50 [1.27-1.78] ***
**Age at CT scan, years**		
45-55	Ref	Ref
56-65	**1.27 [1.10-1.48] *****	**1.55 [1.19-2.01]*****
≥66	**1.79 [1.53-2.09] ***	**2.45 [1.89-3.18] ***
**Education level**		
High	Ref	Ref
Moderate	1.06 [0.94-1.21]	1.15 [0.94-1.41]
Low	**1.18 [1.00-1.40] *****	**1.35 [1.06-1.72]*****
**SHS exposure**		
No	Ref	Ref
Yes	0.99 [0.87-1.13]	1.01 [0.82-1.25]
**Alcohol use**		
No drinking/mild	Ref	Ref
Moderate	0.99 [0.86-1.14]	0.99 [0.79-1.24]
Heavy	1.05 [0.91-1.20]	0.99 [0.81-1.23]
**Asbestos exposure**		
No	Ref	Ref
Yes	1.20 [0.94-1.51]	1.06 [0.76-1.48]
**Body Mass Index**		
Normal	Ref	Ref
Overweight/obesity	0.97 [0.87-1.09]	**0.83 [0.70-0.99]*****
**Physical activity**		
High	Ref	Ref
Low	1.00 [0.89-1.12]	0.99 [0.83-1.18]
**Family history of lung cancer**		
No	Ref	Ref
Yes	0.95 [0.79-1.14]	0.85 [0.64-1.13]
**Cardiovascular disease**		
No	Ref	Ref
Yes	0.98 [0.71-1.33]	0.79 [0.50-1.25]
**Diabetes**		
No	Ref	Ref
Yes	0.98 [0.71-1.36]	0.87 [0.54-1.40]
**COPD**		
No	Ref	Ref

Yes	1.12 [0.99-1.28]	1.11 [0.92-1.33]
**Allergy**		
No	Ref	Ref
Yes	1.06 [0.93-1.21]	0.93 [0.76-1.15]

ORs in former smoker were adjusted by age, sex, educational level, SHS exposure, alcohol use, asbestos exposure, Body Mass Index, physical activity, family history of lung cancer, cardiovascular disease, diabetes, COPD, allergy.

Abbreviations: SHS, secondhand smoke; OR, odds ratio; 95%CI, 95% Confidence Interval.

*P value <0.001, ** P values<0.05

Supplementary Table S5: Multivariable logistic regression analysis of the relationship between population characteristics and lung nodules in current smokers (N=1622)

	**Lung nodules (volume ≥ 30mm^3)**	**Clinically relevant lung nodules (volume ≥ 100mm^3)**
	**OR [95%CI]**	**OR [95%CI]**
**Sex**		
Female	Ref	Ref
Male	**1.36 [1.10-1.69] ****	**1.45 [1.07-1.98] ****
**Age at CT scan, years**		
45-55	Ref	Ref
56-65	**1.34 [1.06-1.69] ****	**1.54 [1.09-2.17]****
≥66	**1.99 [1.46-2.70] ***	**2.00 [1.30-3.02] ****
**Education level**		
High	Ref	Ref
Moderate	1.16 [0.89-1.50]	1.35 [0.91-2.00]
Low	**1.55 [1.14-2.12] ****	**1.63 [1.04-2.54]***
**SHS exposure**		
No	Ref	Ref
Yes	1.03 [0.83-1.29]	0.82 [0.61-1.12]
**Alcohol use**		
No drinking/mild	Ref	Ref
Moderate	0.82 [0.62-1.08]	0.77 [0.52-1.16]
Heavy	1.01 [0.79-1.30]	0.93 [0.65-1.33]
**Asbestos exposure**		
No	Ref	Ref
Yes	1.30 [0.83-2.04]	**1.95 [1.14-3.35]****
**Body Mass Index**		
Normal	Ref	Ref
Overweight/obesity	0.81 [0.65-1.00]	**0.71 [0.52-0.97]****
**Physical activity**		
High	Ref	Ref
Low	1.19 [0.96-1.48]	**1.49 [1.08-2.06]****
**Family history of lung cancer**		
No	Ref	Ref
Yes	1.08 [0.75-1.56]	1.26 [0.76-2.09]
**Cardiovascular disease**		
No	Ref	Ref
Yes	1.14 [0.55-2.39]	0.89 [0.33-2.42]
**Diabetes**		
No	Ref	Ref
Yes	1.33 [0.50-3.49]	0.84 [0.23-3.05]
**COPD**		
No	Ref	Ref

Yes	1.18 [0.94-1.47]	1.06 [0.77-1.44]
**Allergy**		
No	Ref	Ref
Yes	0.99 [0.76-1.28]	1.16 [0.80-1.68]

ORs in current smoker were adjusted by age, sex, educational level, SHS exposure, alcohol use, asbestos exposure, Body Mass Index, physical activity, family history of lung cancer, cardiovascular disease, diabetes, COPD, allergy.

Abbreviations: SHS, secondhand smoke; OR, odds ratio; 95%CI, 95% Confidence Interval.

*P value <0.001, ** P values<0.05

Supplementary Table S6 Summary of the management pathway for lung nodules.

Protocol	Year	Management pathway					
		Nodule type	Nodule size	Count	Risk of malignancy	Recommendation	
**ACCP[1]**	2013	Solid	≤4mm		Low risk	Optional follow-up	
					High risk	Follow-up CT at 12 M	
			4-6mm			Follow-up CT at 6-12 M, and again at 18-24 M	
			6-8mm			Follow-up CT at 3-6 M, at 9-12 M, and again at 18-24 M	
			>8mm		Low risk <5%[&]	Follow-up CT at 3-6 M, at 9-12 M, and again at 18-24 M	
					Moderate risk (5-65%)[&]	PET/CT and optional biopsy/ resection	
					High risk > 65%[&]	Staging for treatment	
		Ground glass	≤5mm			No routine follow-up indicated	
			>5mm			Annual screening for at least 3 years	
		Part-solid (>50%	≤8mm			Follow-up CT at 3, 12 and 24 M, and annual screening for 1 to 3 years	
		Ground glass)	>8mm			Follow-up CT at 3 M, and PET/CT and optional biopsy/ resection	
**BTS[2]**	2015	Solid	<5 mm (<80mm^3)			No routine follow-up	
			5-8mm			5-6mm at 1 Y follow-up CT	1) 2 Y stable on diameter /1 Y stable on volume /VDT>600
						6-8mm at 3 M follow-up CT	D: discharge
			≥8mm (≥300mm^3)		< 10% risk*		2) VDT 400-600 D: biopsy /further follow-up
					>10% risk*	< 10% risk[#]	3) VDT < 400 D: definitive management
						10-70% risk[#]	Biopsy/ further follow-up
						>70% risk[#]	Definitive management
		Subsolid	<5 mm			No routine follow-up	
			≥5mm			Follow-up CT at 3 M	1) Resolved: discharge
							2) Stable: < 10% risk*: CT at 1,2, 4Y;
							>10% risk*: definitive management
							3) Growth: definitive management

**Fleischner Society guideline[3]**	2017	Solid	<6mm (<100mm^3)	Single/ Multiple	Low/ High risk	No routine follow-up indicated<br>Optional CT at 12 months	
			6-8mm (100-250 mm^3)	Single	Low risk	Follow-up CT at 6-12 M, then consider CT at 18-24	
					High risk	Follow-up CT at 6-12 M, then CT at 18-24	
				Multiple	Low risk	Follow-up CT at 3-6 M, then consider CT at 18-24	
					High risk	Follow-up CT at 3-6 M, then CT at 18-24	
			>8mm (≥250mm^3)	Single	All	Consider follow-up CT at 3M, PET/CT or Biopsy	
				Multiple	Low risk	Follow-up CT at 3-6 M, then consider CT at 18-24	
					High risk	Follow-up CT at 3-6 M, then CT at 18-24	
		Ground glass	<6mm (<100mm^3)			No routine follow-up indicated	
			≥6mm (>100mm^3)			Follow-up CT at 6-12 M to confirm persistence, then CT at 3 and 5 years	
		Part-solid	<6mm (<100mm^3)			No routine follow-up indicated	
			≥6mm (>100mm^3)			Follow-up CT at 3-6 M to confirm persistence, then annual CT for 5 years	
		Multiple subsolid	<6mm (<100mm^3)			Follow-up CT at 3-6 M. If stable CT at 2 and 4 years	
			≥6mm (>100mm^3)			Follow-up CT at 3-6 M. Subsequent management based on most suspicious nodule	
**EUPS[4]**	2017	Solid	<100mm^3 (<5 mm)			Next round screening	Management according to category at 3 M
			100-300mm^3 (5-10mm)			Follow-up CT at 3 M	1) VDT > 600 D: Next round screening<br>2) VDT 400-600 D: second repeat scan at 3 M<br>3) VDT≤600D: definitive management
			≥300mm^3 (≥10mm)			Definitive management	
**NCCN [5]**	2022	Solid	<6 mm			Annual screening, until patient is no longer a candidate for definitive treatment	
			6-8mm			Follow-up CT at 6 M	
			8-15mm			Follow-up CT at 3 M, OR consider PET/CT	
			≥15mm			CT+ contrast and/or PET/CT	Low suspicion: follow-up CT at 3 M<br>High suspicion: biopsy/ resection
		Subsolid	<20 mm			Annual screening, until patient is no longer a candidate for definitive treatment	

≥20mm	Follow-up CT at 6 M

ACCP= American College of Chest Physicians; BTS= British Thoracic Society; EUPS= European Union Position Statement; NCCN=National Comprehensive Cancer Network; CT= computed tomography; PET-CT= positron emission tomography; Y, year; M, month; D, day; VDT= volume doubling time

* The Brock model was used to assess the risk of malignancy on follow CT. # The Herder model was used to reassess the malignancy risk in nodules that are evaluated with PET-CT.

& The clinical probability of malignancy was calculated based on the clinical and radiographic characteristics.

References

1. Gould MK, Donington J, Lynch WR, et al. Evaluation of individuals with pulmonary nodules: when is it lung cancer? Diagnosis and management of lung cancer, 3rd ed: American College of Chest Physicians evidence-based clinical practice guidelines. *Chest* 2013: 143(5 Suppl): e93S-e120S.
2. Callister ME, Baldwin DR, Akram AR, et al. British Thoracic Society guidelines for the investigation and management of pulmonary nodules. *Thorax* 2015: 70 Suppl 2: ii1-ii54.
3. MacMahon H, Naidich DP, Goo JM, et al. Guidelines for Management of Incidental Pulmonary Nodules Detected on CT Images: From the Fleischner Society 2017. *Radiology* 2017: 284(1): 228-243.
4. Oudkerk M, Devaraj A, Vliegenthart R, et al. European position statement on lung cancer screening. *The Lancet Oncology* 2017: 18(12): e754-e766.
5. Wood DE, Kazerooni EA, Baum SL, et al. Lung Cancer Screening, Version 3.2018, NCCN Clinical Practice Guidelines in Oncology. *J Natl Compr Canc Netw* 2018: 16(4): 412-441.
