# Supplementary material for: Who is at risk of lung nodules on low-dose CT in a Western country? A population-based approach
Source: Eur Respir J. 2024 Jun 6;63(6):2301736. doi: 10.1183/13993003.01736-2023 (PMC11154756; doi:10.1183/13993003.01736-2023)
Supplement: Supplementary file 2 [file ERJ-01736-2023.Shareable.pdf]

Who is at risk of lung nodules on low-dose CT in a Western country? A population-based approach

Jiali Cai, Marleen Vonder, Yihui Du, Gert Jan Pelgrim, Mieneke Rook, Gerdien Kramer, Harry J.M. Groen, Rozemarijn Vliegenthart and Geertruida H. de Bock

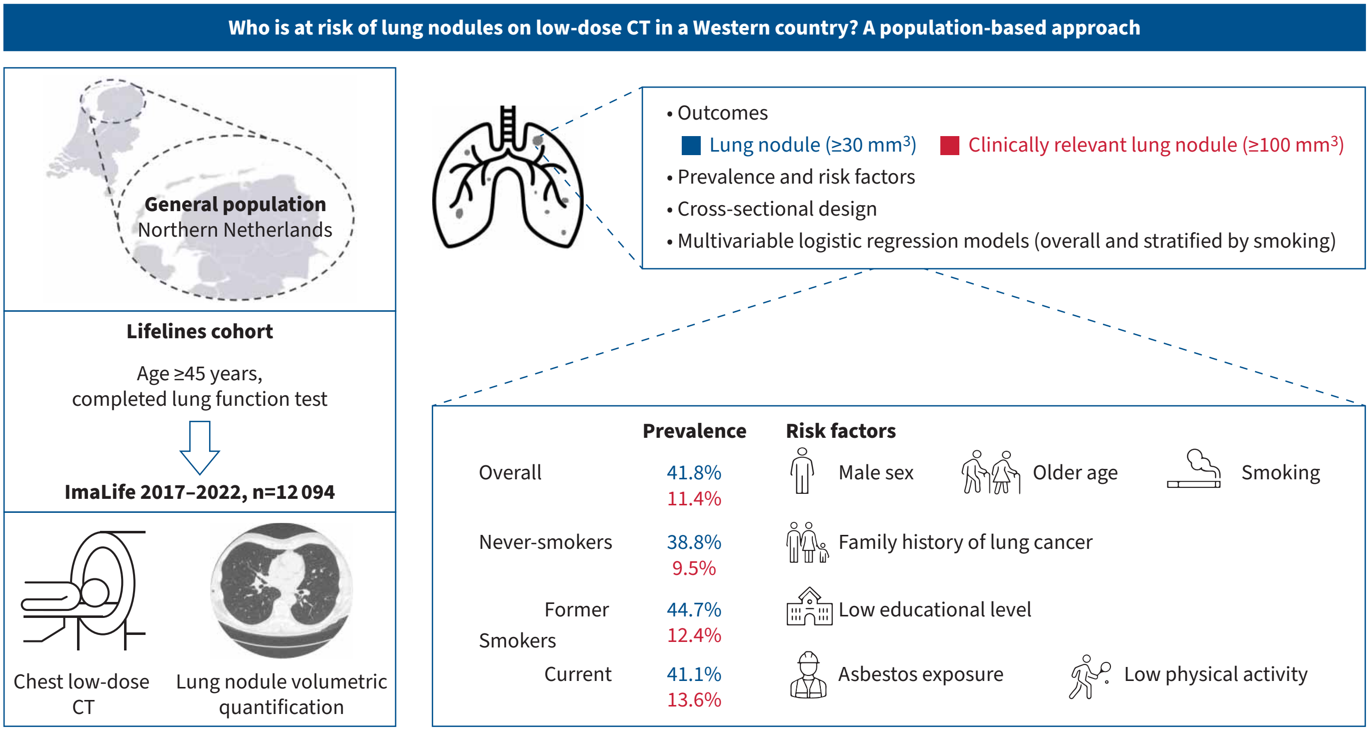

GRAPHICAL ABSTRACT Overview of the risk factors associated with the presence of lung nodules in a Western European general population comprising current smokers, former smokers and never-smokers. CT: computed tomography.

EUROPEAN RESPIRATORY JOURNAL
ORIGINAL RESEARCH ARTICLE
J. CAI ET AL.

SHAREABLE PDF

Who is at risk of lung nodules on low-dose CT in a Western country? A population-based approach

Jiali Cai [1], Marleen Vonder[1], Yihui Du [1,2], Gert Jan Pelgrim [3,4], Mieneke Rook [5], Gerdien Kramer[3,5], Harry J.M. Groen [6], Rozemarijn Vliegenthart [3] and Geertruida H. de Bock [1]

[1]Department of Epidemiology, University of Groningen, University Medical Center Groningen, Groningen, The Netherlands. [2]Department of Epidemiology and Health Statistics, School of Public Health, Hangzhou Normal University, Hangzhou, China. [3]Department of Radiology, University of Groningen, University Medical Center Groningen, Groningen, The Netherlands. [4]Department of Radiology, Medisch Spectrum Twente, University of Twente, Enschede, The Netherlands. [5]Department of Radiology, Martini Hospital Groningen, Groningen, The Netherlands. [6]Department of Pulmonology, University of Groningen, University Medical Center Groningen, Groningen, The Netherlands.

Corresponding author: Geertruida H. de Bock (g.h.de.bock@umcg.nl)

Shareable abstract (@ERSpublications)
Lung nodules were prevalent in a Western European general population and risk factors associated with nodule presence may help to select the most suitable individuals/subgroups for screening and optimise screening eligibility criteria https://bit.ly/3W0JBPy

Cite this article as: Cai J, Vonder M, Du Y, *et al*. Who is at risk of lung nodules on low-dose CT in a Western country? A population-based approach. *Eur Respir J* 2024; 63: 2301736 [DOI: 10.1183/13993003.01736-2023].

This extracted version can be shared freely online.

Copyright ©The authors 2024.

This version is distributed under the terms of the Creative Commons Attribution Licence 4.0.

This article has an editorial commentary: https://doi.org/10.1183/13993003.00889-2024

Received: 10 Oct 2023
Accepted: 12 April 2024

Abstract

Background This population-based study aimed to identify the risk factors for lung nodules in a Western European general population.

Methods We quantified the presence or absence of lung nodules among 12 055 participants of the Dutch population-based ImaLife (Imaging in Lifelines) study (age ⩾45 years) who underwent low-dose chest computed tomography. Outcomes included the presence of 1) at least one solid lung nodule (volume ⩾30 mm^3) and 2) a clinically relevant lung nodule (volume ⩾100 mm^3). Fully adjusted multivariable logistic regression models were applied overall and stratified by smoking status to identify independent risk factors for the presence of nodules.

Results Among the 12 055 participants (44.1% male; median age 60 years; 39.9% never-smokers; 98.7% White), we found lung nodules in 41.8% (5045 out of 12 055) and clinically relevant nodules in 11.4% (1377 out of 12 055); the corresponding figures among never-smokers were 38.8% and 9.5%, respectively. Factors independently associated with increased odds of having any lung nodule included male sex, older age, low educational level, former smoking, asbestos exposure and COPD. Among never-smokers, a family history of lung cancer increased the odds of both lung nodules and clinically relevant nodules. Among former and current smokers, low educational level was positively associated with lung nodules, whereas being overweight was negatively associated. Among current smokers, asbestos exposure and low physical activity were associated with clinically relevant nodules.

Conclusions The study provides a large-scale evaluation of lung nodules and associated risk factors in a Western European general population: lung nodules and clinically relevant nodules were prevalent, and never-smokers with a family history of lung cancer were a non-negligible group.
